# Characterization of the complete mitochondrial genome of *Lysmata debelius* (Decapoda: Hippolytidae)

**DOI:** 10.1080/23802359.2021.1942269

**Published:** 2021-06-23

**Authors:** Jinhui Chen, Changhua Xian, Yuehong Luo, Manfen Lin

**Affiliations:** a Qingyuan Polytechnic, Qingyuan, China; b Guangdong Polytechnic of Science and Trade, Guangzhou, China

**Keywords:** Mitochondrial genome, phylogeny, Hippolytidae

## Abstract

Despite its wide distribution in the Indo–Pacific region and its popularity as an aquarium species, genetic studies on *Lysmata debelius* remain very limited. In this study, we obtained and characterized the complete mitochondrial genome (mitogenome) of *L. debelius*. Results showed that the mitogenome was 16,757 bp in length and consisted of 37 genes, including 13 protein-coding genes (PCGs), 22 transfer RNA genes, and two ribosomal RNA genes. Fourteen genes were light-strand encoded and 23 genes were heavy-strand encoded. The A + T content of the heavy strand was 67.16%. All PCGs had ATN as the start codon. Eight PCGs terminated with a complete stop codon of TAN, and five PCGs (ND3, ND5, ND4, Cox2, and Cytb) had an incomplete stop codon. The phylogeny of 42 shrimp species showed that all three *Lysmata* species were clustered together. The newly described mitogenome should provide valuable data for phylogenetic analysis of Hippolytidae.

The fire shrimp *Lysmata debelius* (Decapoda: Hippolytidae) is a popular ornamental aquarium species with a bright red body covered in several white spots (Hettiarachchi and Edirisinghe 2016). It is widely distributed in the Indo–Pacific region, including along the coasts of Indonesia, Sri Lanka, Japan, and the Philippines (Chace 1997). To date, however, only limited research on its breeding, larval development, and feeding has been reported (Hettiarachchi and Edirisinghe 2016; Simoes et al. 2002). Genetic markers from mitochondrial DNA are highly effective for studies on population genetics, molecular phylogenetics, and evolution (Desalle et al. 2017). At present, however, only partial 16S and 12S sequences of *L. debelius* are available in GenBank. Thus, in this study, we investigated the complete mitochondrial genome (mitogenome) of *L. debelius* and its phylogenetic relationships with other shrimp species.

Specimens (voucher no. QP20200615-1) were collected from the Huangsha Aquatic Products Market in Guangzhou (23°11′ N, 113°25′ E), Guangdong Province, China, and were stored in the herbarium at Qingyuan Polytechnic, Qingyuan City, Guangdong Province, China. The person in charge of the collection is Jinhui Chen (jhchenqypt@126.com). Muscle samples of *L. debelius* were collected, dissected, and preserved at −80 °C until use. The muscle tissue was then used for mitochondrial DNA (mtDNA) extraction with a TIANamp Marine Animals DNA Kit (Tiangen, Beijing, China) according to the manufacturer’s specifications. MtDNA was sequenced using the Illumina Hiseq Sequencing System (Illumina Inc., San Diego, CA, USA). Clean data were acquired and assembled using SPAdes v3.15.2 (Bankevich et al. 2012). MITO (http://mitos.bioinf.uni-leipzig.de/index.py) (Bernt et al. 2013) and ORF Finder (https://www.ncbi.nlm.nih.gov/orffinder/) were used to identify and annotate the protein-coding genes (PCGs), transfer RNA (tRNA) genes, and ribosomal RNA (rRNA) genes. Phylogenetic analysis was conducted using maximum-likelihood (ML) in MEGA v6.0 (Tamura et al. 2013).

The mitogenome of *L. debelius* was 16,757 bp in length (GenBank accession number: MW691200) and included 13 PCGs, 22 tRNA genes, and two rRNA genes. Of the 37 genes, 23 were encoded by the heavy strand, and 14 were encoded by the light strand [including four PCGs (ND1, ND4, ND4L, and ND5), two rRNA genes, and eight tRNA genes]. The contents of A, T, G, and C of the heavy strand were 33.04, 34.12, 13.15, and 19.70%, respectively, with a high A + T content of 67.16%. All PCGs had ATN as the start codon. Seven PCGs (ND1, ND2, ND4L, ND6, Cox1, Cox3, and ATP6) contained a TAA stop codon, one PCG (ATP8) contained a TAG stop codon, and five PCGs (ND3, ND5, ND4, Cox2, and Cytb) contained an incomplete T-stop codon. The 16S and 12S rRNAs were 1338 and 829 bp in length, respectively. All tRNA genes ranged from 64 to 71 bp in size.

Based on the 13 complete concatenated PCGs from 42 shrimp species obtained from GenBank, a phylogenetic tree was constructed using the ML method (Figure 1). The phylogenetic tree showed that the Penaeidae family belonged to Dendrobranchiata and was clustered with several species of Pleocyemata, consistent with previous studies (Kim et al. 2019). The three species of *Lysmata* (i.e. *L. debelius*, *Lysmata amboinensis*, and *Lysmata vittata*) were clustered together. In conclusion, we described the complete mitogenome of *L. debelius* and analyzed its phylogenetic position. The results obtained in the current study should contribute to future phylogenetic studies and population genetic analyses for *L. debelius*.

**Figure 1.**
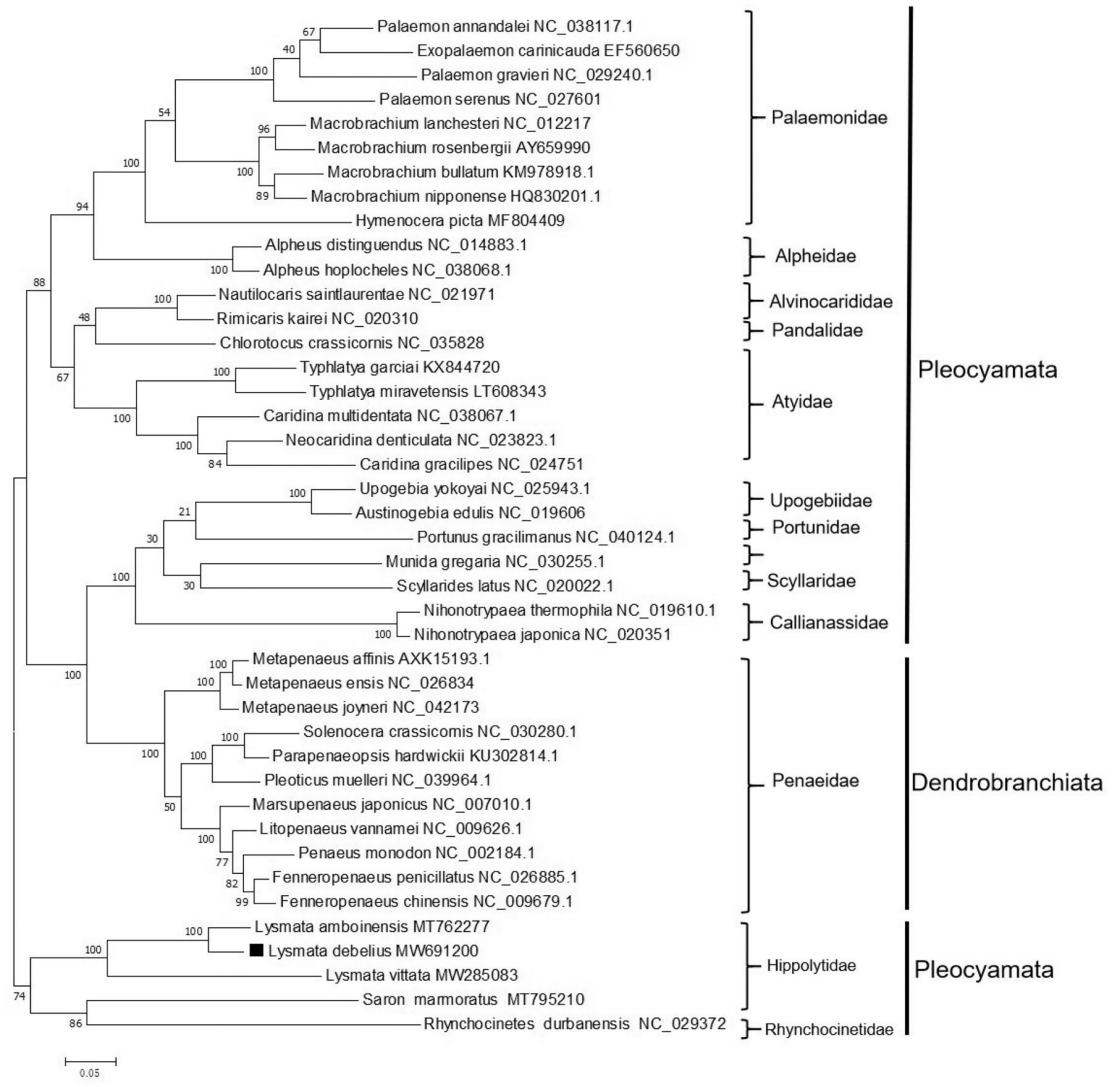
Phylogenetic tree of *Lysmata debelius* and related species based on maximum likelihood (ML) method.

## Data Availability

The data that support the findings of this study are openly available in NCBI at https://www.ncbi.nlm.nih.gov/, reference number MW691200. Associated BioProject, SRA, and BioSample accession numbers are https://www.ncbi.nlm.nih.gov/bioproject/PRJNA720760, https://www.ncbi.nlm.nih.gov/sra/PRJNA720760, and https://www.ncbi.nlm.nih.gov/biosample/SAMN18679091, respectively.
